# Patents on Endophytic Fungi for Agriculture and Bio- and Phytoremediation Applications

**DOI:** 10.3390/microorganisms8081237

**Published:** 2020-08-14

**Authors:** Humberto E. Ortega, Daniel Torres-Mendoza, Luis Cubilla-Rios

**Affiliations:** 1 Laboratory of Tropical Bioorganic Chemistry, Faculty of Natural, Exact Sciences and Technology, University of Panama, Panama 0824, Panama; humberto.ortegad@up.ac.pa (H.E.O.); dtorresm.507@gmail.com (D.T.-M.); 2 Department of Organic Chemistry, Faculty of Natural, Exact Sciences and Technology, University of Panama, Panama 0824, Panama; 3 Vicerrectoría de Investigación y Postgrado, University of Panama, Panama 0824, Panama

**Keywords:** endophytic fungi, patent, abiotic stress tolerance, biocontrol, bioremediation, phytoremediation

## Abstract

Plant endophytic fungi spend all or part of their lives inside host tissues without causing disease symptoms. They can colonize the plant to protect against predators, pathogens and abiotic stresses generated by drought, salinity, high concentrations of heavy metals, UV radiation and temperature fluctuations. They can also promote plant growth through the biosynthesis of phytohormones and nutrient acquisition. In recent years, the study of endophytic fungi for biological control of plant diseases and pests has been intensified to try to reduce the ecological and public health impacts due the use of chemicals and the emergence of fungicide resistance. In this review, we examine 185 patents related to endophytic fungi (from January 1988 to December 2019) and discuss their applicability for abiotic stress tolerance and growth promotion of plants, as agents for biocontrol of herbivores and plant pathogens and bio- and phytoremediation applications.

## 1. Introduction

An endophytic fungus is any organism inhabiting plant organs that, at certain point in its lifetime, can colonize tissues without causing apparent harm [1]. Endophytic fungi have been a proven source of secondary metabolites with potential uses as anticancer, antibiotics, antivirals, anti-inflammatories, antioxidants, neuroprotective agents, insecticides and antifungals, and have multiple applications in biotechnological developments in pharmaceutical, agriculture, cosmetic, food industry and environmental processes [2]. In the last decades, studies of endophytic fungi have resulted in a number of patents linked to the production of biologically active secondary metabolites and in biotransformation processes [3].

Moreover, interaction between fungi and their hosts drives changes in the host metabolism, altering the response to environmental stress and predator attack. Additionally, this interaction leads to the production of secondary metabolites by both the fungi and the host, which further enhance the capability to respond to the environment [4,5,6,7].

The use of endophytic fungi for environmental applications such as growth promotion, relief of abiotic stress, biocontrol of pest and plant pathogens and bio/phytoremediation has gained important attention in recent years due to the concern about global climate change and contamination in soils and natural sources that increases stress in crops, limiting and reducing the production [8,9,10,11]. Furthermore, basic and applied research has been conducted to develop processes, methodologies and technologies that resulted in a considerable number of patents with new proposals to overcome some of these challenges. Therefore, in this review, we cover patents on endophytic fungi applications related to (a) abiotic stress tolerance and growth promotion of plants; (b) biocontrol of herbivores and plant pathogens; (c) bio- or phytoremediation.

The highlighted topics in each of the patents, cited here, could inspire other researchers to take their investigation to the next level and contribute to overcome, in a more efficient way, some of the principal challenges of humanity today.

## 2. Materials and Methods

The present review was conducted mainly through searches in the Scifinder^®^ and Google Patents databases. The search was initially conducted in Scifinder^®^ using the terms “endophytic fungi” and “patents” covering the period from 1988 to 2019. 12,315 references were found. After removing duplicates (those describing the same patent/endophyte), we selected those related to the aim of this review, resulting in 185 documents. The patents covered in this study are described in five tables below. 

## 3. Results

The description and analysis of patents was divided, considering the main objective of each one, into four sections; those associated to: (Section 3.1) abiotic stress tolerance and growth promotion of plants; (Section 3.2) biocontrol of herbivores and plant pathogens, and (Section 3.3) bio- and phytoremediation applications; (Section 3.4) patents where the endophyte has multiple applications. The information in tables describe the fungi, the host plant where they were isolated, and the main application of the patent. All endophytes, listed in the tables, have beneficial effects on plants, even though some of them could be considered as pathogens in previous reports.

### 3.1. Abiotic Stress Tolerance and Growth Promotion of Plants

The principal abiotic stress factors in plants include drought, salinity, high heavy metal concentrations, UV radiation and temperature fluctuations [12]. Abiotic stress affects the cellular pathways of plants, resulting in negative changes to their physiology and morphology [12]. Endophytic fungi have been shown to help their host plant to overcome abiotic stress and promote plant growth through the biosynthesis of phytohormones (indole-3-acetic-acid, gibberellins, cytokinins, ethylene, acetoin, 2, 3-butanediol) and nutrient absorption and uptake [12,13,14].

Plant endophytic fungi have been patented based on their ability to improve the following in plants: (a) root and seed development; (b) nutrient uptake or absorption; (c) photosynthesis promotion; (d) growth of biomass; (e) increase chlorophyll content; and (f) abiotic stress resistance. Numerous genera have been used for such purposes, including *Acremonium*, *Alternaria*, *Aspergillus*, *Chaetomium*, *Fusarium*, *Penicillium,* and others (Table 1 and Table 2). A specific area of application for which endophytic fungi have been widely used is in the growth promotion of medicinal plants; this includes such species as *Acanthopanax senticosus* [15], *Salvia miltiorrhiza* [16], *Rumex gmelinii* Turcz [17], *Acacia confusa* [18], *Coix lacryma-jobi* [19], *Cynanchum acuminata* [20], *Huperzia serrata* [21], *Anoectochilus roxburghii* [22], *Arnebia* sp. [23], *Saussurea* sp. [24], *Rhizoma bletillae* [25], *Salvia miltiorrhiza* [26,27], and *Eucalyptus* sp. [28,29,30]. Additionally, some endophytic fungi have been patented due to their capability to promote the growth of crop plants such as corn, tomato, soybean, rice, wheat, potato, and barley [31,32,33,34,35,36,37] as well as other useful plants such as *Casuarina equisetifolia* [38,39,40,41], fir [42,43,44,45], *Aleurites montana* [46,47,48,49,50,51], *Dendrobium* sp. [52,53,54], tobacco [55,56,57,58], *Schima superba* [59,60,61], *Bletilla striata* [62,63], and *Paphiopedilum* sp. [64].

### 3.2. Biocontrol of Herbivores and Plant Pathogens

Crop plant diseases represent a major threat in agriculture [101]. The number of chemicals that can be effectively used to control pathogens has been reduced due to the emergence of fungicide resistance along with an increased awareness of the negative associated ecological and public health impacts [101]. Due to these problems, study of the biological control of plant diseases with endophytes has intensified in recent years [101]. Endophytes have been shown to protect their hosts against diseases, reducing infection levels and inhibiting the growth of pathogens [102,103]. The proposed mechanisms used by endophytes are the production of antimicrobial and structural compounds, niche competition, and the induction of plant immunity [104].

Several patents describe the biocontrol of herbivores and plant pathogens using endophytic fungi (Table 3). Species of the genus *Acremonium* have been described to control *Verticillium* wilt [105]; Argentine stem weevil (*Listronotus bonariensis*) [106]; plant diseases caused by banana root nematode and different pathogenic microbes such as *Bipolaris oryzae*, *Colletotrichum falcatum*, *Colletotrichum gloeosporioides*, *Corynespora cassiicola*, *Corynespora* sp., *Drechslera* sp., *Fusarium oxysporum*, *Gloeosporium musarum*, and *Magnaporthe grisea* [107]; and to prevent fescue toxicosis [108]. Species of *Alternaria* can control the growth of different pathogens such as *Rhizoctonia solani*, *Fusarium oxysporum*, *Botrytis cinerea*, *Phytophthora capsici*, *Pseudomonas aeruginosa*, *Proteus hauseri*, and *Plasmopara viticola* [109,110,111,112,113,114,115]. Members of the genus *Aspergillus* have been applied to limit the growth of nematodes in soil [116]; the plant pathogenic fungi *Sclerotinia sclerotiorum*, *Rhizoctonia solani,* and *Thanatephorus cucumeris* [52,117,118]; as well as grass fungi [119]. Several strains of the genus *Chaetomium* have been reported to enhance plant disease resistance in *Anoectochilus roxburghii* cultivation [16], to control different plant pathogenic fungi [120,121,122], to inhibit *Erwinia* causing soft rot and *Ralstonia solanacearum* causing bacterial wilt [123], to inhibit anthracnose apple pathogens [124], in the preparation of an anti-plant pathogen fermentation liquid broth [125], and in the production of chaetoglobosin A with antagonistic activity against *Exserohilum turcicum*, *Coniothyrium diplodiella,* and *Rhizopus stolonifer* [126]. Species of *Fusarium* can prevent and treat black spot and fungal diseases in *Panax notoginseng* [127,128], control five plant pathogenic fungi (*Fusarium oxysporum*, *Cytospora mandshurica*, *Colletotrichum gloeosporioides*, *Venturia pyrina*, and *Fusarium graminearum*) [129], and control rice blast disease [130,131] and bacterial wilt of ginger [132]. Species of *Neotyphodium* can decrease the mildewing rate of *Elymus sibiricus* seeds at the germination stage [133] and improve fungicide and pest resistance in plants [134,135]. Species of *Penicillium* can restrain the effects of *Panax notoginseng* anthracnose, root rot [136,137,138], and *Alternaria panax* [139]; control different harmful pathogenic fungi [140,141] and litchi downy blight [142]; and prevent plant diseases such as *Sclerotinia* rot of colza and tobacco blackleg [53]. Species of *Rhexocercosporidium* can control the fungal pathogens *Colletotrichum gloeosporioides*, *Fusarium solani*, and *Alternaria panax* Whetzel on *Panax notoginseng* [143,144,145].

Endophytic fungi of different genera such as *Beauveria*, *Cladosporium*, *Metarhizium*, *Muscodor*, *Trichoderma,* and others have also been described in patents to control pests or different plant diseases (Table 3).

### 3.3. Bio- and Phytoremediation

Bioremediation is a process that uses microorganisms, plants or enzymes to detoxify contamination in natural sources. In phytoremediation, plants and their own metabolic system can extract toxic chemicals from water, soil and air. This chemicals or contaminants include metals and metalloid pollutants, carcinogenic agents, industrial organic waste material, inorganic pesticides and herbicides, chlorinated products, excess nutrients and radionuclides [10,11,192].

**Figure 1:**
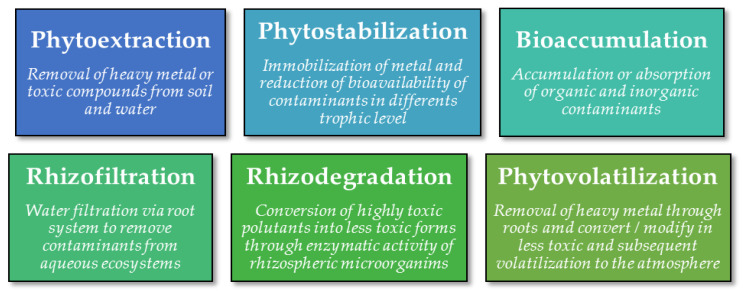
Bio- and phytoremediation approaches involving endophytic fungi.

Endophytic fungi have the capability to degrade small and large organic compounds by enzymatic reactions, decompose environmental contaminants, and improve the soil microenvironment [193]. They can also increase the ability of host plants to remove contaminants from soil, water, sediment, and air [194], and to modulate morphological and physiological functions in the host plant improving its resistance to metals and providing different detoxification routes such as extracellular scavenging and complexation, compartmentalization and volatilization [14,195]. Figure 1 shows different bioremediation techniques involving endophytic fungi.

Some patents describe the use of endophytic fungi for bioremediation and phytoremediation (Table 4). Strains of the genus *Fusarium* have been reported to induce phytoremediation in heavy metal-contaminated soil [196], repair uranium-polluted water bodies [197], and decontaminate and decompose human and animal waste [198]. Additionally, the endophytic fungi Y2R14 and RWDL4-1 can be used to treat wastewater polluted by cadmium [199]. Heavy metals such as mercury, cadmium, arsenic, chromium, and lead are toxic at low concentrations. They can be accumulated in the ecosystem inside living organisms and are capable of entering the food chain [200]. The functions of several organs of the human body can be affected by heavy metals, and some of these substances can cause cancer by long-term exposure [200]. Uranium is a radioactive substance and is also harmful for the environment and human beings [197]. The use of microorganisms to repair large areas of farmland pollution can reduce costs, the use of large amounts of chemicals, and secondary pollution [196].

Species of *Phomopsis* and *Xylaria* have been reported to degrade the herbicide MCPA (2-methyl-4-chlorophenoxyacetic acid) in water and soil [201,202]. Additionally, several genera of fungi can be used to produce high-laccase content for soil bioremediation [203].

### 3.4. Patents that Claim Multiple Applications

A small number of patents comprised more than one possible application (Table 5); this is the case of the applications for *Neotyphodium uncinatum* to induce insect resistant and drought tolerance in plants [204]; *Phoma* sp. can improve salt stress resistance, promote the growth and increase biomass in crop plants such as wheat and rice [205]; *Clonostachys rosea* promotes plant growth, stress resistance and reduces dependency on chemical pesticides [206,207]; *Fusarium* sp. stimulates plant growth and reduces heavy metal absorption in tobacco [208], and *Rhizoctonia* sp. fosters plant growth and stress resistance in *Anoectochilus roxburghii* [22].

We found two patents, whose applications implicated the use a plural number of fungi (genus/species); one of them claims the capability to increase biomass and promote biotic and abiotic stress resistance in cereal crops [37], the other claims to improve dry shoot weight, mean dry grain weight and suppression of seed-borne in cereal crops [35].

## 4. Discussion

**Figure 2:**
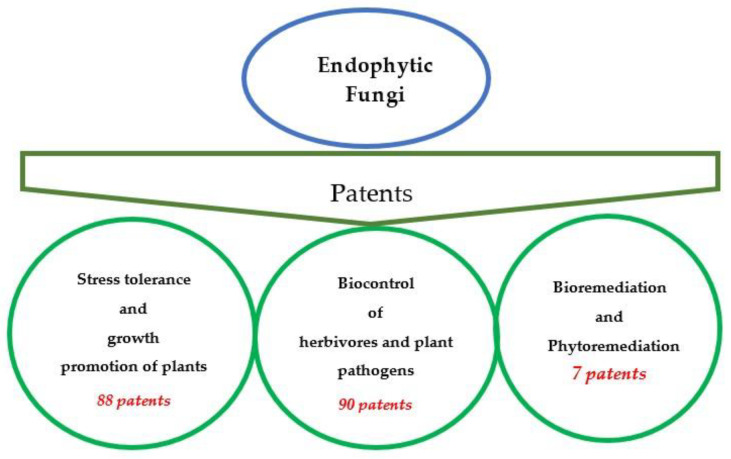
Total number of patents for area of application in the period 1988 to 2019.

In the present review, we highlight a wide number of endophytic fungi that have been patented for developing processes, methodologies, or new techniques in applications that include but are not restricted to (a) alternatives to overcome biotic and abiotic stress and to reduce the use of chemicals associated with environmental toxicity in agricultural practices, (b) the degradation of harmful compounds, and (c) improvement in the ability of plants to remove contaminants from soil, water, and air. Abiotic stress tolerance and growth promotion of plants, and biocontrol of herbivores and plant pathogens, were the most patentable applications of endophytic fungi with 88 and 90 patents, respectively; concerning bio- and phytoremediation, 7 patents were recorded for the period 1988–2019 (Figure 2). The most representative genera of these applications belong to *Alternaria*, *Aspergillus*, *Chaetomium*, *Fusarium*, *Penicillium* and *Muscodor*.

**Figure 3:**
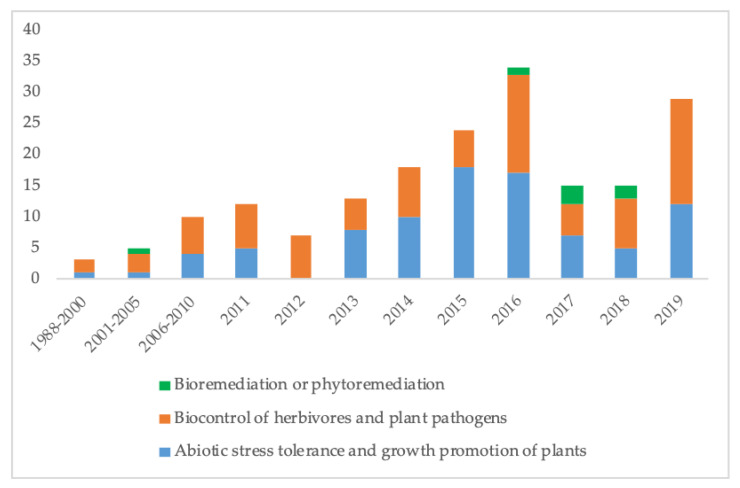
Patents of endophytic fungi for agricultural purposes and bio/phytoremediation registered from 1988 to 2019.

Studies of endophytic fungi ecology have allowed the understanding of the multiple interactions they develop with their host, other endophytes, as well with herbivores and pathogens that put the host under abiotic stress. Nonetheless, it is evident that one individual or group of endophytes can be used for mitigation stresses from different origins. Due to the concerns about global climate change and its implications in food security, there are an increased interest to develop applications for the use of endophytic fungi in abiotic stress tolerance and growth promotion of important food crops [209], as well as the use for biocontrol of herbivores and plant pathogens. This increment can be noted since 2011 as shown in Figure 3. The loss of growing areas due to contamination and the recovery of spaces contaminated by heavy metals, organic and inorganic compounds will lead the focus of research on endophytic fungi for bio- and phytoremediation applications.

Considering the abundance of endophytic fungi under study, the development of patentable applications like those reviewed here, and other applications still unexplored like fungal pigments [210], has become a prominent research area for this class of microorganisms.

### Future Perspectives

The use of endophytic fungi to improve the nutrients absorption in plants can change the optimum usage of organic and inorganic fertilizers [211]. The capability of endophytic fungi to increase biotic and abiotic stress tolerance in plant hosts is an unexplored area for agricultural purposes; the control of pests and diseases under climate change conditions [211]; studies in fungal species related to develop resistance to changes in their environment could lead their application in food production in limited resources areas and as an important alternative for crop production for human sustainability. Many endophytes are now often recognized as symbionts with unique and intimate interactions with the plant host [10]. The genetic engineering of fungi is an easier process than in plants. The genetic modification of endophytic fungi with useful genes could contribute, with new traits, to the inoculation of plants [212].

The use of endophytic fungi on remediation of contaminated ecosystems is an interesting prospect for further studies. Fungi that could increase the capacity of CO_2_ absorption by plants, degradation and biotransformation of waste, enhance food production without altering its quality or those that provided drought resistance/nutrient absorption capability to plant species related to human or animal feeding could be areas of significance to develop new applications and patents. The investigations applied in these fields are forwarded by the advance in the techniques used for the characterization of endophytic fungi and also by the technological advances in analytical techniques for carrying out studies of chemical processes at the cellular level.

## Figures and Tables

**Table 1:** Endophytic fungi applied to enhance the abiotic stress tolerance of plants.

Patent No.	Endophyte	Host ^1^	Patent Application	Ref.
CN104762216A	*Arthrinium* sp.	*Salicornia bigelovii*	Plant anti-salt stress.	[65]
WO2004000017A2	*Curvularia* sp.	*Dichanthelium languinosum*	Conferring stress tolerance to inoculated plants (monocots and dicots).	[66]
WO2009012480A2	*Fusarium* sp.	*Leymus mollis*	Conferring stress tolerance to inoculated plants (monocots and dicots).	[67]
CN105296359A	*Lecanicillium* sp.	Tobacco	Reducing the absorption of heavy metals in tobacco.	[58]
CN101314760A	*Neotyphodium chisosum*	*Festuca arundinacea*	Improving the stress tolerance to drought and diseases.	[68]
CN104004665A	*Papulospora* sp.	Fir roots	Relieving phosphorus stress in fir.	[43]
CN105002099A	*Paraconiothyrium cyclothyrioides*	*Myricaria* root	Reducing heavy metal pollution in plants.	[69]
CN101974437A	*Penicillium* sp.	*Eucalyptus*	Relieving aluminum toxicity in *Eucalyptus*.	[30]
CN102002463A	*Penicillium* sp.	*Eucalyptus* roots, stems, and leaves	Improving the cold resistance of *Eucalyptus*.	[28]
CN103865806A	*Phialophora oryzae*	Not disclosed	Reducing the absorption of heavy metals in tobacco	[57]
CN107926549A	*Piriformospora indica*	Not disclosed	Improving the resistance of plants to the herbicide bensulfuron-methyl.	[70]
CN103834578A	*Pyrenochaeta* sp.	Tobacco	Promoting plant growth and reducing the heavy metal content in tobacco.	[55]
CN105316240A	*Rhizopycnis* sp.	Tobacco	Reducing the absorption of heavy metals in tobacco.	[56]
US20150366217A1	Group of several fungi ^2^	Roots of *Triticum turgidum* L.	Improving seed vitality, biotic and abiotic stress resistance, and plant health and yield under both stressed and unstressed environmental conditions.	[71]

^1^ Some patents just provided a common name for the host organism. ^2^ A list of the group of fungi is in Table S1.

**Table 2:** Endophytic fungi applied for the growth promotion of plants.

Patent No.	Endophyte	Host ^1^	Patent Application	Ref.
CN105907648A	*Acremonium* sp.	*Panax notoginseng*	Root and seed development of different plants including *Radix Ginseng*, *Oryza sativa L.*, *Semen Maydis*, *Semen Tritici aestivi*, *Rhizoma Paridis*, Rhizoma *Solani tuberosi*, etc.	[34]
CN108513990A	*Alternaria alternata*	*Acanthopanax senticosus*	Seedling-stage growth of *A. senticosus*.	[15]
CN104911108A	*Alternaria* sp.	*Hippophae* sp.	Drought resistance on turf grass.	[72]
CN104818218A	*Alternaria* sp.	*Aleurites montana*	Phosphorus uptake in *A. montana*.	[47]
CN102086439A	*Alternaria tenuissima*	*Panax ginseng*	Growth of corn plant.	[31]
CN103173362A	*Aspergillus* sp.	*Casuarina* sp. rhizosphere	Photosynthesis in *C. equisetifolia*.	[38]
CN103173361A	*Aspergillus* sp.	*Casuarina* sp. rhizosphere	Nutrient element absorption in *Casuarina*.	[39]
CN103173364A	*Aspergillus* sp.	*Casuarina* sp. rhizosphere	*Casuarina* biomass growth.	[41]
CN110343619A	*Botryosphaeria* sp.	Root of *Schima superba*	*Schima superba* seedling height and ground diameter under a low-phosphorus environment.	[61]
CN109456902A	*Byssochlamys spectabilis*	*Rhizoma bletillae*	The growth of *R. bletillae*.	[25]
CN106929436A	*Cercosporella Sacc.*	*Rumex gmelini* Turcz	Growth in *R. gmelinii* Turcz.	[17]
CN106801014A	*Chaetomium globosum*	*Salvia miltiorrhiza*	Radix root biomass, plant height, crown diameter in *S. miltiorrhiza*.	[73]
CN109628322A	*Chaetomium nigricolor*	*Bletilla striata*	The growth of *B. striata*.	[62]
CN110438011A	*Cladosporium tenuissimum*	*Salvia miltiorrhiza*	Synthesis of effective components (tanshinone and salvianolic acid substances) in the root system of *Salvia miltiorrhiza*.	[26]
CN104630073A	*Claviceps* sp.	*Dendrobium officinale*	Growth and yield in *D. officinale*.	[74]
CN104004664A	*Colletotrichum* sp.	*Abies* sp. roots	Photosynthesis of cedar.	[45]
CN106085872A	*Colletotrichum* sp./*Fusarium* sp.	*Acacia* sp.	Nutrient absorption in *A. confusa*.	[18]
CN104805019A	*Coniothyrium* sp.	*Aleurites* sp.	Nutrient element absorption in wood oil tree.	[75]
CN104004666A	*Cylindrocarpon* sp.	fir plant	Growth of fir.	[42]
CN110250210A	*Darksidea* sp.	*Stipa capillata* root	Rooting and growth of maize.	[36]
CN109504611A	*Diaporthe spectabilis*	*Bletilla striata*	Growth of *B. striata*.	[63]
CN103733829A	*Emericella foeniculicola*	*Salvia miltiorrhizae*	Growth of *S. miltiorrhizae*.	[76]
CN105624047A	*Epichloë bromicola*	*Coix lacryma-jobi*	Growth of *Coix lacryma-jobi*, *Arabidopsis thaliana* and other graminaceous plants.	[19]
CN105861334A	*Filobasidium* sp.	*Acacia* sp.	Taiwan *Acacia* biomass.	[77]
CN105861335A	*Filobasidium* sp.	*Acacia* sp.	Nutrient element absorption in Taiwan *Acacia* in a low-phosphorous environment.	[78]
CN106085873A	*Filobasidium* sp./*Penicillium* sp.	*Acacia* sp.	Phosphorous uptake in *A. confusa* under a low-phosphorus environment.	[79]
CN107432135A	*Fusarium redolens*	Not disclosed	Germination of *Cynanchum acuminata* seeds.	[20]
CN103173360A	*Fusarium* sp.	*Casuarina equisetifolia*	Chlorophyll content of *C. equisetifolia*.	[80]
CN110257259A	*Fusarium* sp.	*Schima superba stems*	Photosynthesis of *Schima superba*.	[59]
CN103114044A	*Heterodera oryzae*	rice	Plant growth regulation and/or plant pathogenicity.	[81]
CN103798293A	*Hypha* sp.	*Salvia miltiorrhiza*	The growth and improvement of *S. miltiorrhiza* hairy root tanshinone content.	[82]
CN1961631A	*Mycocentrospora* sp./*Leptodontidium* sp.	*Saussurea involucrata*	*Saussurea* sp. growth.	[24]
CN104593274A	*Nectria* sp.	*Dendrobium officinale*	Yield in *Dendrobium* artificial planting.	[83]
US20130104263A1	*Neotyphodium* sp.	perennial ryegrass	Beneficial properties (phenotype) for plant.	[84]
CN104004667A	*Paecilomyces* sp.	Not disclosed	Phosphorus absorption in fir.	[44]
CN106010984A	*Penicillium* sp.	*Acacia confusa*	Plant biomass growth of Taiwan *Acacia* plant under low-phosphorus environment.	[85]
CN101974438A	*Penicillium* sp.	*Eucalyptus*	Phosphorus absorption in *Eucalyptus*.	[29]
CN104818219A	*Penicillium* sp.	*Aleurites montana*	Root growth of *A. montana* in a low-phosphorous environment.	[51]
CN104789481A	*Penicillium* sp.	*Aleurites montana*	Growth and photosynthesis enhancement of *A. montana* in a low-phosphorus environment.	[48]
CN104762219A	*Penicillium* sp.	*Aleurites montana*	Biomass growth of *A. montana* in a low-phosphorus environment.	[49]
CN110257258A	*Penicillium* sp.	*Schima superba* leaves	Phosphorus absorption of *Schima superba*.	[60]
WO2016210238A1	*Penicillium* sp.	Not disclosed	Cultivation of agricultural plants, such as soybean and maize.	[33]
CN104818217A	*Pestalotia* sp.	Not disclosed	Biomass growth of *A. montana*.	[50]
CN105886405A	*Pestalotiopsis* sp.	*Dendrobium officinale*	Growth of *D. officinale* and change in metabolic components.	[54]
CN107988087A	*Pezicula ericae*	wild blueberry root	Growth effects.	[86]
CN109706084A	*Phoma herbarum*	*Salvia miltiorrhiza*	Growth of *Salvia miltiorrhiza* and synthesis of tanshinone compounds.	[27]
CN104593273A	*Phyllachora* sp.	*Dendrobium officinale*	*Dendrobium* yield.	[87]
CN103173363A	*Phyllosticta* sp.	*Casuarina* sp.	Photosynthesis of *C. equisetifolia*.	[40]
ES2500790A1	*Pochonia chlamydosporia*	Not disclosed	Flowering and fruiting and increased yield in crops such as tomatoes.	[32]
WO2016038234A1	*Pochonia chlamydosporia*	*Meloidogyne* spp.	Culture yield and reduction in flowering and fructification times.	[88]
CN105039172A	*Pythium* sp.	*Huperzia serrata*	Improved transplant survival rate of *H. serrate*.	[21]
CN108041078A	*Rhizopycnis* sp.	tobacco	Rice growth.	[89]
WO2019113255A1	*Serendipita vermifera* ssp. *bescii*	Australian orchid	Enhancement of plant performance in combination with phosphite as a phosphorous source.	[90]
CN105420119A	*Schizophyllum commune*	*Ginseng*	Host tissue culture hairy root biomass and ingredients of ginseng saponins.	[91]
CN104774771A	*Thermomyces* sp.	Not disclosed	Photosynthesis of *A. montana* under a low-phosphorus environment.	[46]
CN107046965A	*Trichoderma* sp.	*Anoectochilus formosanus*	Seedling adaptation cultivation.	[92]
CN104745482A	*Trichoderma* sp.	*Arnebia euchroma*	Growth of *Arnebia* hairy roots and improved shikonin component content in hairy roots.	[23]
CN105969672A	*Trichoderma* sp. *Fusarium* sp.	*Acacia* sp.	Increase in the height and ground diameter of *A. confusa* seedlings.	[93]
CN110408551A	*Tulasnella calospora*	Roots of *Paphiopedilum*	Growth of aseptic seedlings of *Paphiopedilum*.	[64]
CN102876584A	*Xylaria striata*	*Oryza meyeriana*	Plant growth.	[94]
CN107460133A	*Zasmidium* sp.	mangrove	Growth and development of *D. officinale*.	[95]
WO2016179047A1	*Group of fungi*	Not disclosed	Agronomic traits in plants.	[96]
CZ306950B6	*Group of fungi*	*Miscanthus* sp.	Growth, especially of graminaceous and *Miscanthus* plants.	[97]
WO2017134664A1	*Acremonium sclerotigenum/Sarocladium implicatum*	Set of grass relatives of wheat	Nutrient uptake.	[98]
US20150373993A1	Group of several ^2^ fungi	A diverse type of wild relatives or ancestral landraces of maize, wheat, rice, and other seeds	Agronomic traits.	[99]
WO2018102733A1	Group of several ^2^ fungi	Agricultural plants	Modulation of the nutritional quality traits in seeds	[100]

^1^ Some patents just provided a common name for the host organism. ^2^ A list of the group of fungi is in Table S1.

**Table 3:** Endophytic fungi applied as biocontrol agents of herbivores and plant pathogens.

Patent No.	Endophyte	Host ^1^	Patent Application	Ref.
CN103897992A	*Acremonium alternatum*	cotton	*Verticillium* wilt.	[105]
US93951A0	*Acremonium coenophialum*	Not disclosed	Fescue toxicosis.	[108]
AU639084B2	*Acremonium lolii*	French perennial ryegrass ecotype	Argentine stem weevil (*Listronotus bonariensis*) by production of compound peramine.	[106]
CN101235355A	*Acremonium strictum*	*Brachiaria brizantha*	Banana root-knot nematode and different pathogenic microbes.	[107]
WO2012174585A1	*Acremonium* sp.	*Brachiaria*/*Urochloa*	Fungal plant diseases.	[146]
CN108192832A	*Acrocalymma* sp.	*Sinomenium acutum*	Plant diseases caused by pathogenic bacteria.	[147]
CN108085259A	*Arcopilus aureus*	*Dendrobium sp.*	The plant pathogenic fungus *Botrytis cinerea.*	[148]
CN102204570A	*Alternaria alternata*	*Cinnamomum camphora*	*Rhizoctonia solani*, *Fusarium oxysporum*, and *Botrytis cinerea.*	[111]
CN102191184A	*Alternaria alternata*	*Cinnamomum camphora*	Plant pathogenic fungi such as *Rhizoctonia solani*, *Fusarium oxysporum,* and *Botrytis cinerea.*	[110]
CN110373331A	*Alternaria alternata*	*Huperzia serrata*	Gray mold of crops.	[115]
ES2696982A1	*Alternaria alternata* and *Fusarium acuminatum*	*Artemisia thuscula* and *Austrian Artemisia*	Plant pathogenic fungi with the production of antifungal compounds.	[114]
CN103232942A	*Alternaria* sp.	*Spiraea* sp.	The plant pathogenic fungus *Phytophthora capsici*.	[112]
CN106520572A	*Alternaria mali*	*Toona sinensis*	The pathogens *Pseudomonas aeruginosa* or *Proteus hauseri*.	[113]
WO2008007251A2	*Alternaria alternata*	Not disclosed	*Plasmopara viticola*.	[109]
CN108441426A	*Aspergillus niger*	Aquatic plant	Plant parasitic nematodes in soil.	[116]
CN104560735A	*Aspergillus oryzae*	*Tephrosia purpurea*	Plant pathogenic fungi such as *Sclerotinia* rot of colza and tobacco black shank disease.	[52]
CN102191185A	*Aspergillus restrictus*	*Allium sativum*	Plant pathogenic fungi such as *Rhizoctonia solani* and *Thanatephorus cucumeris.*	[117]
CN109504610A	*Aspergillus* sp.	Epiphyte	The pathogenic fungus *rhizoctonia solani.*	[118]
CN108342328A	*Aspergillus versicolor*	seaweed	Grass fungi.	[119]
US8709399B2	*Beauveria bassiana*	maize stem borer *Busseola fusca*	Herbivorous insects and/or plant pathogens.	[149]
CN105462892A	*Burkholderia* sp.	*Sophora tonkinensis*	*Panax notoginseng* black spot.	[150]
CN105838613A	*Chaetomium globosum*	*Cajanus cajan*	Fungal plant diseases with the production of flavipin.	[151]
CN107475123A	*Chaetomium globosum*	*Anoectochilus roxburghii*	Plant disease in *Anoectochilus roxburghii* cultivation.	[16]
CN102742605A	*Chaetomium globosum*	*Ginkgo biloba*	Plant pathogenic fungi.	[122]
CN102690759A	*Chaetomium globosum*	*Solidago canadensis*	Plant pathogenic fungi propagation	[121]
CN101280320A	*Chaetomium globosum*	Not disclosed	Plant fungal diseases with the production of antibiotic substances	[120]
CN106754396A	*Chaetomium globosum*	*Toona sinensis*	*Erwinia* and *Ralstonia solanacearum*	[123]
CN104877919A	*Chaetomium globosum*	*Phellopterus littoralis*	Anthracnose pathogens of apples and certain inhibitory actions against other plant pathogens	[124]
CN103255065A	*Chaetomium globosum*	*Camptotheca acuminata*	Plant pathogens with broth culture of the endophytic fungi	[125]
CN102754652A	*Chaetomium globosum*	*Ginkgo biloba*	*Exserohilum turcicum*, *Coniothyrium diplodiella*, and *Rhizopus stolonifer*	[126]
CN105368720A	*Chaetomium* sp.	Healthy cotton plant	Cotton *Verticillium* wilt.	[152]
CN109749938A	*Cladosporium tenuissimum*	Healthy *Panax notoginseng*	*Panax notoginseng* rot.	[153]
CN110172408A	*Clonostachys rosea*	*Podophyllum hexandrum*	Diseases and pests of *Podophyllum hexandrum*.	[154]
CN110272829A	*Colletotrichum boninense*	*Huperzia serrata*	*Sclerotinia sclerotiorum* of crops.	[155]
WO2014136070A1	*Epichloë*	*Elymus mutabilis*	Pests on *Secale* spp. plants.	[156]
CN105483022A	*Fusarium solani*	*Sophora tonkinensis*	*Panax notoginseng* black spot.	[127]
CN105483021A	*Fusarium solani*	*Sophora tonkinensis*	*Panax notoginseng* fungal diseases.	[128]
CN103194490A	*Fusarium solani*	*Ginkgo biloba*	Five plant pathogenic fungi.	[129]
CN105087386A	*Fusarium* sp.	Yinchuan *Phragmites communis*	Rice blast disease.	[130]
CN108624527A	*Fusarium* sp.	*Ginkgo sp.*	Bacterial wilt in ginger.	[132]
CN110558337A	*Fusarium oxysporum*	*Ginkgo biloba*	Rice blast disease.	[131]
CN102174416A	*Fusella* sp.	*Angelica sinensis*	Plant pathogenic bacteria.	[157]
WO2016034751A1	*Guignardia mangiferae*	*Persea indica*	Phytopathogens and plant pests.	[158]
WO2013081448A2	*Hendersonia* sp.	Not disclosed	Basal stem rot disease and *Ganoderma* disease in oil palms.	[159]
CN109536390A	*Hypoxylon* sp. nov	Midvein of citrus leaves	Citrus black spot disease.	[160]
CN103642704A	*Leptosphaeria* sp.	cotton	Cotton *Verticillium* wilt.	[161]
CN103289906A	*Metarhizium* sp.	*Gentiana manshurica*	*G. manshurica* leaf blight.	[162]
CN110229758A	*Mortierella elongata*	*Atractylodes macrocephala*	*Atractylodes macrocephala* root rot.	[163]
CN101691541A	*Muscodor sp.*	Not disclosed	Pathogenic fungi.	[164]
US20040141955A1	*Muscodor albus and Muscodor roseus*	Not disclosed	Organisms such as microbes, insects, and nematodes with volatile compounds.	[165]
WO2002082898A1	*Muscodor albus and Muscodor roseus*	Not disclosed	Plant pathogens, bacteria, nematodes, and insects with volatile antibiotics.	[166]
WO2010115156A2	*Muscodor strobelii*	Not disclosed	Pests and pathogenic microbes, including *Ganoderma boninense.*	[167]
WO2004034785A2	*Muscodor vitigenus*	*Paullinia paullinioides*	Insects with the production of repellents by a novel endophytic fungus.	[168]
CN106893678A	*Myrothecium verrucaria*	grapes	Grape gray mold.	[169]
CN104774768A	*Nectria haematococca*	*Fritillaria wabuensis*	Bacteria such as *S. aureus* and *P. aeruginosa* and pathogenic fungi.	[170]
CN106538108A	*Neotyphodium sp.*	gramineous plants	Mildewing rate of *Elymus sibiricus* seeds in the germination stage.	[133]
WO2007021200A1	*Neotyphodium* sp.	Not disclosed	Plant pathogenic fungi.	[134]
CA2319847C	*Neotyphodium* sp.	*Festuca arundinacea*	Pests and reduce ergopeptine alkaloid levels.	[135]
CN102191186A	*Nigrospora oryzae*	*Allium sativum*	Plant pathogenic fungi such as *Rhizoctonia solani*, *Colletotrichum lindemuthianum*, and *Botrytis cinerea*.	[171]
CN104789482A	*Nigrospora sp.*	*Magnolia officinalis*	Wheat disease.	[172]
CN110178857A	*Paecilomyces variotii*	*Hippophae rhamnoides*	Plant virus. Induces plant endogenous salicylic acid accumulation and enhances the plant RNA silencing efficiency.	[173]
CN105462854A	*Penicillium citrinum*	*Sophora tonkinensis*	*Panax notoginseng* anthracnose.	[136]
CN105462850A	*Penicillium citrinum*	*Sophora tonkinensis*	*Panax notoginseng* root rot.	[137]
CN105462855A	*Penicillium citrinum*	*Sophora tonkinensis* Gagnep	*Alternaria panax*.	[139]
CN104531543A	*Penicillium griseofulvum*	*Tephrosia purpurea*	Plant diseases such as *Sclerotinia* rot of colza, tobacco blackleg, and others with a fermentation product.	[53]
CN105255742A	*Penicillium* sp.	*Malus hupehensis*	Harmful pathogens such as *Fusarium solani*, *F. proliferatum*, *F. moniliforme,* and *F. oxysporum.*	[140]
CN108546651A	*Penicillium* sp.	*Kandelia candel*	Plant pathogenic fungi such as *Fusarium graminearum*, *Phytophthora sojae,* and *Colletotrichum musae* with a fermentation product.	[141]
CN109112069A	*Penicillium* sp.	*Panax notoginseng* root	*Panax notoginseng* root rot.	[138]
CN103773699A	*Penicillium purpurogenum*	Litchi	Litchi downy blight.	[142]
CN103627643A	*Penicillium simplicissimum*	Healthy cotton plant	Cotton *Verticillium* wilt.	[174]
CN104161049A	*Pestalotiopsis uvicola*	*Artemisia japonica*	Kiwifruit *Sclerotinia sclerotiorum*, *Phytophthora capsici,* and other plant pathogenic fungi with a fermentation product.	[175]
CN110511878A	*Pezicula neosporulosa*	Fir	The pathogenic fungus *Fusarium oxysporum.*	[176]
CN109769535A	*Phialophora oryzae*	Wild rice root	Bacterial blight of rice.	[177]
CN102154116A	*Phomopsis wenchengensis*	Not disclosed	Plant pathogenic fungi by antifungal compounds.	[178]
CN105462853A	*Rhexocercosporidium* sp.	*Sophora tonkinensis*	*Colletotrichum gloeosporioides* on *Panax notoginseng*.	[143]
CN105462851A	*Rhexocercosporidium* sp.	*Sophora tonkinensis*	*Fusarium solani* on *Panax notoginseng.*	[144]
CN105462848A	*Rhexocercosporidium* sp.	*Sophora tonkinensis*	*Alternaria panax* Whetzel on *Panax notoginseng.*	[145]
CN102234618A	*Rhizopus* and *Trichoderma*	Not disclosed	Soft rot disease of the orchid family *Dendrobium* plants.	[179]
CN110452290A	*Sarocladium brachiariae*	*Brachiaria brizantha*	Plant disease and pests.	[180]
CN110468057A	*Seimatosporium* sp.	*Rosa multiflora*	Tobacco powdery mildew caused by *Erysiphe cichoracearum.*	[181]
CN106167767A	*Schizothecium* sp.	Not disclosed	Banana wilt.	[182]
CN110558336A	*Spirillum roseum*	Not disclosed	Lettuce *sclerotinia* rot.	[183]
CN103834580A	*Talaromyces flavus*	Not disclosed	Cotton *Verticillium* wilt	[184]
CN106119134A	*Talaromyces flavus*	Not disclosed	Fruit rot	[185]
CN109593658A	*Talaromyces* sp.	*Fructus corni*	Fungal diseases of wheat	[186]
CN105211105A	*Trichothecium roseum*	strawberries	Powdery mildew of wheat	[187]
US20120108425A1	*Trichoderma atroviride*	healthy tea leaves	Foliar disease in tea plantations caused by *Cercospora theae*	[188]
CN108179115A	*Zopfiella* sp.	*Chrysanthemum morifolium*	Plant pathogens such as *Fusarium moniliforme*, *F. oxysporum*, *Curvularia lunata*, and *Pythium*	[189]
WO2018119419A1	Group of several ^2^ fungi	cotton	Nematodes, aphids, flea hopper, lygus bug, stink bug, soy looper, cabbage looper, or fungi	[190]
US9469836B2	Not disclosed	*Pinus strobus*	Pests in *Pinus strobus*	[191]

^1^ Some patents just provided a common name for the host organism. ^2^ A list of the group of fungi is in Table S1.

**Table 4:** Endophytic fungi applied in bioremediation and phytoremediation.

Patent No.	Endophyte	Host ^1^	Patent Application	Ref.
CN105733958A	*Fusarium oxysporum*	Not disclosed	Phytoremediation of heavy metal-contaminated soil	[196]
CN106340337A	*Fusarium* sp.	mangrove	Repair of uranium-polluted water body	[197]
WO2005116272A2	*Fusarium culmorum* and *Muscodor albus*	Not disclosed	Decontamination and decomposition of human and animal waste	[198]
CN106947697A	*Phomopsis* sp.	Not disclosed	Degradation of the herbicide MCPA (2-methyl-4-chlorophenoxyacetic acid) in water or soil	[201]
CN107177511A	*Xylaria* sp.	Not disclosed	Degradation of the herbicide MCPA in water and soil	[202]
CN107900098A	Group of several fungi ^2^	Not disclosed	Production and application of a high-laccase content soil remediation agent	[203]
CN108751424A	Not disclosed	wild soybean	Treatment of wastewater polluted by the heavy metal cadmium	[199]

^1^ Some patents just provided a common name for the host organism. ^2^ A list of the group of fungi is in Table S1.

**Table 5:** Patents that claim multiple applications.

Patent No.	Endophyte	Host ^1^	Patent Application	Ref.
WO2000062600A1	*Neotyphodium uncinatum*	meadow fescue	Import desired traits: include no adverse effects on herbivore, insect resistance, drought tolerance and improved persistence in the plants.	[204]
CN104293681A	*Phoma* sp.	Not disclosed	Improving salt stress resistance in rice and wheat. Promotion of growth in rice seedling, delaying salt damage of wheat in saline and alkaline land. Increasing biomass accumulation in wheat.	[205]
US20160007613A1	*Clonostachys rosea*	Not disclosed	Promotion of plant vigor, health, growth, yield, and resistance to competitive stress.	[206]
WO2007107000A1	*Clonostachys rosea*	Not disclosed	Enhanced plant vigor, health, growth, yield, reducing environmental stress and reduction of dependency on chemical pesticides for pest control.	[207]
CN103849572A	*Fusarium* sp.	Not disclosed	Promoting plant growth and reduction of heavy metal absorption in tobacco.	[208]
CN101953261A	*Rhizoctonia* sp.	*Anoectochilus roxburghii*	Growth of *A. roxburghii*, improved the reproductive rate, survival rate and stress resistance.	[22]
WO2019115582A1	Group of several fungi ^2^	*Hordeum murinum*	Increased yield and biomass in cereal crops, and promotes biotic and abiotic stress resistance in cereal crops	[37]
WO2016030535A1	*Group of several fungi* ^2^	*Hordeum murinum subsp. murinum*	Improving dry shoot weight, mean dry grain weight and suppression of seed-borne infection in a cereal crop.	[35]

^1^ Some patents just provided a common name for the host organism. ^2^ A list of the group of fungi is in Table S1.

## Supplementary Materials

The following are available online at https://www.mdpi.com/2076-2607/8/8/1237/s1, Table S1: List of patens grounded in the use of several endophytic fungi to develop applications.
