# Supplementary material for: Patents on Endophytic Fungi for Agriculture and Bio- and Phytoremediation Applications
Source: Microorganisms. 2020 Aug 14;8(8):1237. doi: 10.3390/microorganisms8081237 (PMC7465599; doi:10.3390/microorganisms8081237)
Supplement: Supplementary file 1 [file microorganisms-08-01237-s001.pdf]

Supplementary Material

Patents on endophytic fungi for agriculture and bio-and phytoremediation applications

Humberto E. Ortega, Daniel Torres-Mendoza and Luis Cubilla-Rios

Table S1. List of patens grounded in the use of several endophytic fungi to develop applications

Patent No.	Endophytic fungi
US20150366217A1	*Paraconyothirium* sp., *Pseudeurotium* sp., *Penicillium* sp., *Cladosporium* sp.
WO2016179047A1	*Acremonium* sp., *Alternaria* sp., *Aspergillus* sp., *Aureobasidium* sp., *Biscogniauxia* sp., *Botryosphaeria* sp., *Botrytis* sp., *Bullera* sp., *Cercospora* sp., *Chaetothriales*, *Cladosporum* sp., *Cochliobolus* sp., *Coniothyrium* sp., *Cryptococcus* spp., *Davidiella* sp., *Dioszegia* sp., *Dothideales*, *Dothideomycetes*, *Epicoccum* sp., *Erysiphe* sp., *Erythrobasidium* sp., *Fusarium* sp., *Gibberella* sp., *Hannaella* sp., *Hormonema* sp., *Hypoxylon* sp., *Lecythophora* sp., *Leptospora* sp., *Lewia* sp., *Monodictys sp.*, *Monographella* sp., *Mucor* sp., *Nectria* sp., *Neurospora* sp., *Paraconiothyrium* sp., *Parastagonospora* sp., *Penicillium* sp., *Periconia* sp., *Pestalotiopsis* sp., *Phaeomoniella* sp., *Phoma* sp., *Phyllosticta* sp., *Pichia* sp., *Pleosporales*, *Preussia* sp., *Rhizopus* sp., *Rhodosporidium* sp., *Sordariomycetes*, *Sporobolomyces*, *Sporormiaceae*, *Stagonospora* sp., *Udeniomyces*, *Wallemia* sp., *Xylaria* sp.
CZ306950B6	*Verticillium leptobactrum*, *Exophiala* sp., *Halenospora* sp., *Zalerion* sp.
US20150373993A1	*Acremonium* sp., *Alternaria* sp., *Cladosporium* sp., *Cochliobolus* sp., *Embelisia* sp., *Epicoccum* sp., *Fusarium* sp., *Nigrospora* sp., *Phoma* sp., *Podospora* sp.
WO2018102733A1	*Curvularia* sp., *Epicoccum* sp., *Periconia* sp., *Acremonium* sp., *Cladosporium* sp., *Exserohilum* sp., *Paraconiothyrium* sp., *Penicillium* sp., Pseudeurotium sp., *Phialemonium* sp.
WO2018119419A1	*Cladosporium* sp., *Sphaerulina* sp., *Epicoccum* sp., *Stagonospora* sp., *Alternaria* sp., *Bipolaris* sp., *Stemphylium* sp., *Preussia* sp., *Curvularia* sp., *Penicillium* sp., *Phomopsis* sp., *Gibellulopsis* sp., *Acremonium* sp., *Fusarium* sp., *Purpureocillium* sp., *Chaetomium* sp., *Cryptococcus* sp.
CN107900098A	*Alternaria* sp., *Cunninghamella* sp., *Penicillium* sp., *Phoma* sp., *Fusarium* sp., *Gilmaniella* sp., *Sclerotium* sp., *Acremonium* sp., *Ceratobasidium stevensii*, *Rhizoctonia* spp., *Pestalotiopsis* spp., *Acremoniella* spp., *Chaetomium* spp., *Coniothyrium* spp., *Coryneum* spp., *Dothiorella* spp., *Phomopsis* sp.
WO2019115582A1	*Alternaria alternata*, *Penicillium brevicompactum*, *Penicillium glabrum*, *Cladosporium ramontenellum*, *Cladosporium herbarum*, *Humicola grisea*, *Talaromyces purpurogenus*
WO2016030535A1	*Paecilomyces marquandii*, *Viridispora alata*, *Cladosporium* sp., *Penicillium brevicompactum*, *Pyronochaeta unguis-hominis*, uncultured *Metarhizium*, uncultured *fungus FJ820798*, *Exophiala oligosperma*
